# The correlation between traditional Chinese medicine constitutions and chronic kidney disease-associated pruritus among hemodialysis patients in Macau

**DOI:** 10.1097/MD.0000000000045952

**Published:** 2025-11-14

**Authors:** Yao-Chen Chuang, Yinghong Zhang, Kun-Han Chuang, Xin Wang, Jianwei Wu

**Affiliations:** a Nursing and Health Education Research Centre, Kiang Wu Nursing College of Macau, Macao 999078, China; b Department of Dialysis Center, Kiang Wu Hospital, Macao, China; c Department of Physiology and Pathophysiology, School of Basic Medical Sciences, Fujian Medical University, Fuzhou, China.

**Keywords:** chronic kidney disease-associated pruritus, deficiency constitution, excess constitution, hemodialysis, traditional Chinese medicine constitution

## Abstract

Traditional Chinese medicine (TCM) constitutions are associated with susceptibility to certain diseases. Studying the types of TCM constitutions associated with diseases helps to predict the occurrence, progression, and prognosis of these diseases. This study is the first to explore the correlation between deficiency constitution, excess constitution, and chronic kidney disease-associated pruritus (CKD-aP). This cross-sectional study was conducted between January and June 2023 at Kiang Wu Hospital. Patients undergoing hemodialysis were interviewed using a questionnaire to collect sociodemographic data and assess pruritus and the TCM constitution. The correlation between body constitution and CKD-aP levels was also analyzed. Of 162 patients analyzed, 78 (48.2%) had a balanced constitution, 49 (30.2%) had a deficient constitution, and 35 (21.6%) had an excess constitution. Both deficiency and excess constitutions were statistically significant in the occurrence of CKD-aP. After adjusting for all possible confounding factors, the analysis showed that compared to the balanced constitution, deficiency constitution (OR = 2.22, 95% CI = 1.025–4.806) and excess constitution (OR = 2.695, 95% CI = 1.091–6.658) significantly increased the likelihood of CKD-aP. A deficient constitution mainly affects the duration, degree, disability, and distribution of pruritus, whereas an excess constitution affects the degree and disability. Both deficiency and excess constitutions were correlated with the occurrence of CKD-aP. Different constitution types affect pruritus differently, necessitating further precise analysis of the interaction between constitution types and pruritus occurrence.

**Key Points**

- Biased constitution is related to the occurrence of chronic kidney disease-associated pruritus.
- Deficiency and excess constitutions affect different dimensions of chronic kidney disease-associated pruritus.

## 1. Introduction

Chronic kidney disease-associated pruritus (CKD-aP) is a common and distressing symptom in patients with end-stage renal disease or those undergoing maintenance hemodialysis. Unlike general pruritus, CKD-aP is persistent and may recur, significantly affecting the patient’s quality of life and is associated with depression, poor sleep quality, and increased mortality.^[1,2]^ Currently, the mechanism underlying the occurrence of CKD-aP is not fully understood. Literature suggests that it may be associated with factors such as xerosis cutis, deposition of uremic toxins in the skin and subcutaneous tissues, immune dysregulation, peripheral neuropathy, dysregulation of the endogenous opioid system, alterations in the skin microbiota, and inflammatory factors.^[3,4]^ Due to the numerous factors associated with CKD-aP and significant individual variability, there is no universal treatment suitable for every patient. Current clinical approaches to alleviate itching include treating xerosis, immunomodulatory therapy, antihistamines, removal of uremic toxins, management of opioid imbalance, peripheral neuropathy therapy, and treatment of hyperparathyroidism. However, the effectiveness of these treatments remains uncertain due to limitations such as single-center, noncontrolled study designs, insufficient sample sizes, and inconsistent definitions of CKD-aP.^[4,5]^ Although difelikefalin – a highly selective κ-opioid receptor agonist – and gabapentinoids, which are γ-aminobutyric acid (GABA) mimetics, have been approved by the U.S. Food and Drug Administration (FDA) and the European Medicines Agency (EMA) and are considered first-line treatments for CKD-aP by clinical experts, further case accumulation is still needed to confirm their therapeutic efficacy.^[6]^ Currently, clinical assessments for pruritus include unidimensional, multidimensional, and other related scales.^[7]^ Since pruritus is a complex sensation that affects patients in various aspects, multidimensional assessments can provide a more comprehensive description of the discomfort caused by itching. The 5D itch scale is a commonly used, single page, brief multidimensional assessment tool that is sensitive to change over time, and not only evaluates the degree, duration, progression, and distribution of pruritus but also assesses its impact on patients’ quality of life.^[8]^ According to surveys from different countries indicate that the prevalence of CKD-aP ranges from 10 to 74%.^[2,9–11]^ These variations may be associated with multiple factors such as race, genetics, environment, and lifestyle. Similarly, these individual and environmental factors also play a role in the development of traditional Chinese medicine (TCM) constitution.^[12]^

Since its establishment in the 1970s, TCM constitution theory has been widely applied in disease diagnosis, prevention, and treatment.^[13]^ TCM constitution theory posits that an individual’s physical condition is influenced by inborn genetic factors and acquired environmental factors, resulting in unique variations among individuals. Based on these differences, constitutions are classified into 9 distinct types including balance, qi deficiency, yang deficiency, yin deficiency, phlegm dampness, damp heat, qi stagnation, blood stasis, and inherited special constitutions. With the exception of the balanced constitution, the other 8 types are considered unbalanced constitutions. These unbalanced constitutions can be further classified into 3 categories: deficiency constitutions, which include qi deficiency, yang deficiency, and yin deficiency constitutions; excess constitutions, which include phlegm dampness, damp heat, blood stasis, and qi stagnation constitutions; special constitutions, referring to inherited special constitution.^[14]^ Different constitution types lead to varying susceptibilities to diseases; therefore, personalized treatment plans tailored to specific constitution types contribute to the realization of precision medicine.^[15,16]^ The most commonly used method for TCM constitution identification is the “ Constitution in Chinese Medicine Questionnaire (CCMQ),” developed by renowned TCM experts such as Wang Qi and officially issued by the China Association of Traditional Chinese Medicine.^[13]^ Previous studies utilizing the CCMQ to investigate the relationship between constitution and disease susceptibility have shown that hypertension and diabetes are often associated with Qi deficiency, Yin deficiency, and Phlegm dampness constitutions; hyperuricemia is linked to Qi deficiency, Phlegm dampness, and Dampness-heat constitutions; gout is frequently related to Phlegm dampness, Dampness-heat, and Blood-stasis constitutions; and insomnia is commonly associated with Qi deficiency, Yang deficiency, Phlegm dampness, and Qi-stagnation constitutions.^[17–19]^ Notably, researchers in Japan have also employed the CCMQ to explore the correlation between TCM constitutions and skin types, and the results supported the “constitution-skin correlation theory” in TCM.^[20]^ In recent years, our team’s research has also found that cognitive impairment tendencies among the elderly in Macau are related to Yin deficiency and Qi-stagnation constitutions.^[21]^ Although numerous disease-constitution correlations have been identified, the constitution types associated with CKD-aP remain unclear. Therefore, utilizing the CCMQ to investigate the susceptibility constitution types related to CKD-aP in hemodialysis patients in the Macao region, combined with the 5D itch scale to analyze the correlations between various itch-related dimensions and constitution categories, will provide a reference basis for developing personalized itching treatment and prevention strategies.

## 2. Materials and methods

### 2.1. Study design and subjects

This study conducted a questionnaire survey among 183 patients undergoing hemodialysis at Kiang Wu Hospital from January to June 2023. The participant selection criteria were as follows: patients receiving maintenance hemodialysis at Kiang Wu Hospital for more than 6 months, with treatment frequency of 3 times per week and 4 hours per session; age > 18 years, with no barriers to verbal or written communication. Patients with pruritus caused by eczema, urticaria, allergic dermatitis, drug-induced rash, etc, as well as those recently using hormones or cytotoxic drugs, were excluded. All participants provided informed consent, and this study was approved by the Ethics Committees of Kiang Wu Hospital and Kiang Wu Nursing College of Macau.

The questionnaire surveyors in this study were full-time nurses in the hemodialysis unit of Kiang Wu Hospital who had previously studied courses in TCM nursing and possessed a background in TCM theory. They also received unified training before conducting the questionnaire interviews. Prior to the interviews, the surveyors explained the significance, purpose, and ethical principles of the study to each participant and obtained written informed consent. Subsequently, the surveyors conducted individual interviews with each participant, clearly reading out each question on the questionnaire for the participants to answer, and recorded the responses in the questionnaire. The sample size was calculated based on the formula: (Zα² × p × q)/ e². Under the conditions of a maximum response distribution rate (*P* = .65, e = 0.07) and a 95% confidence level (z = 1.96), this study required 142 samples. Considering the nonresponse rate, the sample size was increased by 20%. Ultimately, 162 subjects were included in this study, meeting the sample size requirement.

### 2.2. Research tools

The questionnaire used in this study consisted of 3 parts: sociodemographic data (gender, education level, marital status, living conditions, employment status, exercise habits), pruritus condition assessed using the Chinese version of the 5D itch scale. This scale is easy to use, with high content validity, high test-retest reliability, and good internal consistency, yielding a Cronbach alpha coefficient of 0.734. The evaluation of pruritus includes duration, severity, direction, disability, and distribution. Each dimension is scored from 1 to 5, with the total score ranging from 5 to 25. Higher scores indicate greater negative impact.^[8]^ A score ≤ 8 was considered no pruritus, 9 to 11 were classified as mild pruritus, 12 to 17 classified as moderate pruritus, 18 to 21 classified as severe pruritus, and ≥ 22 as very severe pruritus.^[7]^ The TCM constitution assessment was conducted using the 60-item CCMQ. This scale has been validated to demonstrate good reliability and validity, with an internal consistency (Cronbach alpha) ranging from 0.7 to 0.8.^[22]^ Patients responded based on their self-perception over the past year using a 1 to 5 scale. The total score for each constitution type was calculated and converted using the following formula: [(original score–number of items)/ (number of items × 4)] × 100. A converted score of ≥ 60 for the balanced constitution and < 30 for all 8 biased constitutions indicates a balanced constitution; a converted score of ≥ 60 for the balanced constitution and < 40 for the 8 biased constitutions indicates a “near-balanced” constitution. Other determinations were made if the criteria were not satisfied. When the conversion score of a certain biased constitution > 40, it is classified as the corresponding biased constitution; 30 to 39 is classified as “tend to” the biased constitution; if the score < 30, it is not judged as the biased constitution.^[23]^ Generally, if a balanced constitution is identified, no biased constitution is present concurrently, whereas multiple biased constitutions can coexist. Although the coexistence of multiple biased constitutions is permitted during constitution assessment, this study only considered the constitution with the highest score as the primary type for analysis. Additionally, due to sample size limitations, individuals rated as “near-balanced” were categorized under the balanced constitution group, while those classified as “tend to” a certain biased constitution were assigned to the corresponding biased constitution category. Among the 8 biased constitution types, those characterized by deficiency or imbalance of yin-yang, leading to functional decline, are classified under deficiency constitutions, which include qi deficiency, yang deficiency, and yin deficiency constitutions. Cases where qi, blood, or body fluids are excessive, accumulate in the body, form pathogenic factors, and obstruct the movement of qi and blood are categorized as excess constitutions, including phlegm dampness, damp heat, blood stasis, and qi-stagnation constitutions. Those with systemic yin-yang disorder or innate physiological deficiencies are classified as inherited special constitution.^[14]^

### 2.3. Statistical analysis

Excel was used to establish a database, and all data were double entered to ensure accurate entry. The basic characteristics of the research subjects were described by counts and proportions, and the Mann–Whitney U test or Kruskal–Wallis test was used for comparisons between different groups. Multivariable logistic regression analysis was used to analyze the relationship between TCM constitutions and 5D itching, with 5D itching as the dependent variable and TCM constitutions as the independent variable. Gender, age, education, marriage, living with others, employment status, and regular exercise were adjusted for in the model. Odds ratios (OR) and 95% confidence intervals (CI) were calculated.

To analyze the impact of deficiency and excess constitutions on 5D itching, the scores of the 5D dimensions (duration, degree, direction, disability, and distribution) were used as dependent variables, and TCM constitutions were used as independent variables. After controlling for gender age, education, marriage, living with others, employment status, and regular exercise, multiple linear regression models were established. Statistical analyses were performed using SPSS (version 28) for Windows. Significance was accepted at a *P*-value < 0.05 for all calculations.

## 3. Results

### 3.1. Sociodemographic characteristics

Of the 183 distributed questionnaires, 168 were valid, with a response rate of 91.8%. Excluding the 6 with inherited special constitution, of the remaining 162 patients, the average age was 65.00 ± 11.97 years. The majority of patients were male (66.7%). The educational level of most participants was middle school or higher (59.3%). Most participants were married (82.1%) and lived with others (87.7%), with a minority exercising regularly (32.1%) or employed (8.0%) (Table 1).

**Table 1:** Sociodemographic characteristics and degree of 5D itching (n = 162).

Variables	Counts	Degree of 5D itching, n (%)			Mean rank	*Z* or *H* value
		1 = No	2 = Mild	3 = Moderate to severe		
Gender						
Male	108	44 (40.7)	44 (40.7)	20 (18.5)	85.94	−1.856†
Female	54	30 (55.6)	18 (33.3)	6 (11.1)	72.61	
Education						
Primary schools or below	66	27 (40.9)	28 (42.4)	11 (16.7)	85.02	−0.861†
Middle school or above	96	47 (49)	34 (35.4)	15 (15.6)	79.08	
Marriage						
Single	29	12 (41.4)	13 (44.8)	4 (13.8)	83.43	−0.266†
Married	133	62 (46.6)	49 (36.8)	22 (16.5)	81.08	
Living with others						
No	20	6 (30.0)	8 (40.0)	6 (30.0)	98.3	−1.862†
Yes	142	68 (47.9)	54 (38.0)	20 (14.1)	79.13	
Employment status						
No	149	71 (47.7)	53 (35.6)	25 (16.8)	80.48	−1.020†
Yes	13	3 (23.1)	9 (69.2)	1 (7.7)	93.19	
Exercise regularly						
No	110	53 (48.2)	41 (37.3)	16 (14.5)	79.14	−1.015†
Yes	52	21 (40.4)	21 (40.4)	10 (19.2)	86.5	
TCM constitutions						
Balance	78	43 (55.1)	27 (34.6)	8 (10.3)	72.53	6.520‡,*
Deficiency constitution	49	19 (38.8)	18 (36.7)	12 (24.5)	89.91	
Excess constitution	35	12 (34.3)	17 (48.6)	6 (17.1)	89.73	

† *Z* value, statistic of Mann–Whitney *U* test.

‡ *H* value, statistic of Kruskal–Wallis test.

* *P* < .05.

### 3.2. Distribution of TCM constitutions among hemodialysis patients

**Figure 1.**
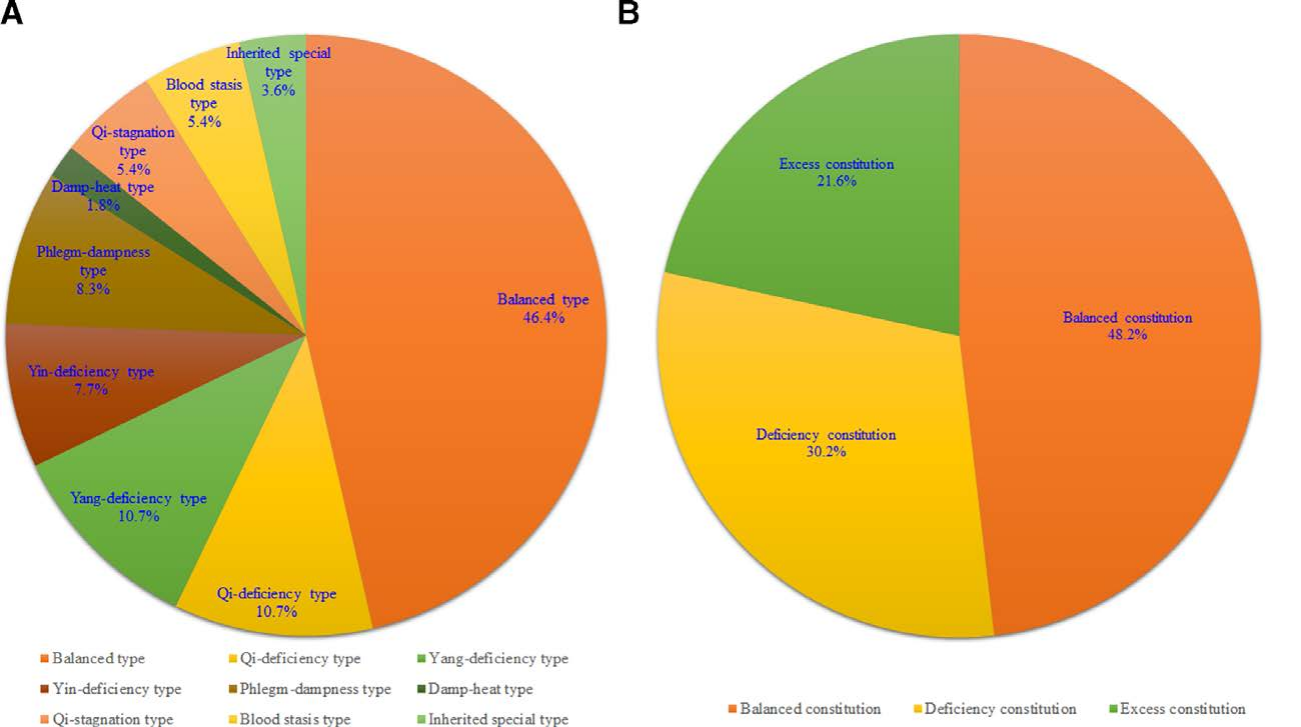
Distribution of TCM constitution among hemodialysis patients. (A) Distribution ratio of people with 9 constitution types. (B) Distribution proportion of people in 3 constitution categories.

Among all 168 valid questionnaires, 78 patients (46.4%) had a balanced constitution; 18 (10.7%) had qi deficiency, 18 (10.7%) had yang deficiency, 13 (7.7%) had yin deficiency, 14 (8.3%) had phlegm dampness, 3 (1.8%) had damp heat, 9 (5.4%) had qi stagnation, 9 (5.4%) had blood stasis, and 6 (3.6%) had inherited a special constitution (Fig. 1A). The inherited special constitution was not classified as deficiency or excess and was therefore excluded from the statistical analysis. Excluding the 6 with inherited special constitution, of the remaining 162 patients, 78 (48.2%) had a balanced constitution and 84 (51.8%) had an imbalanced constitution. Among these, 49 (30.2%) had a deficiency constitution (qi deficiency, yang deficiency, and yin deficiency), and 35 (21.6%) had an excess constitution (phlegm dampness, damp heat, qi stagnation, and blood stasis) (Fig. 1B).

### 3.3. Degree of 5D itching among participants

Among all the 162 participants, more than half (54.3%) of these hemodialysis patients experienced CKD-aP according to the 5D itch scale, with 62 (38.3%) experiencing mild and 26 (16.0%) experiencing moderate to severe itching. Statistical differences (*P* < .05) were observed in the degree of 5D itching among the 3 TCM constitution categories (Table 1).

### 3.4. The association of TCM constitutions and CKD-aP

Hierarchical logistic regression analysis (Table 2) revealed that both deficient (*P* = .043, OR = 2.22, 95% CI = 1.025–4.806) and excess constitution (*P* = .032, OR = 2.695, 95% CI = 1.091–6.658) were positively associated with CKD-aP after adjusting for all potential confounders. The adjusted model successfully classified 67.3% of the cases (R2N = 0.177), indicating that participants with either a deficiency or excess constitution were more likely to experience CKD-aP.

**Table 2:** The logistic regression analysis for 5D itching (n = 162).

Variables	Model 1		Model 2	
	OR (95% CI)	*P*-value	OR (95% CI)	*P*-value
Constant	2.081	.560	1.345	.820
Gender (female = 1)	0.462 (0.221–0.964)	.040	0.414 (0.193–0.887)	.023
Age	1.01 (0.976–1.046)	.567	1.013 (0.977–1.1050)	.489
Education (middle school or above = 1)	0.607 (0.296–1.247)	.174	0.604 (0.287–1.269)	.183
Marriage (married = 1)	0.933 (0.319–2.73)	.899	0.819 (0.264–2.54)	.730
Living with others (Yes = 1)	0.417 (0.134–1.304)	.133	0.397 (0.122–1.29)	.124
Employment status (Yes = 1)	3.451 (0.86–13.851)	.081	3.196 (0.776–13.164)	.108
Exercise regularly (Yes = 1)	1.291 (0.631–2.642)	.485	1.325 (0.631–2.782)	.457
Deficiency constitution (Yes = 1)	–	–	2.22 (1.025–4.806)	.043
Excess constitution (Yes = 1)	–	–	2.695 (1.091–6.658)	.032

### 3.5. The correlation of deficiency and excess constitutions with 5D itching dimensions

Table 3 shows the scores of the 5D itching dimensions in the different categories of TCM constitution. One-way ANOVA indicated significant differences (*P* < .05) in the duration, disability, and distribution dimensions between deficiency and balanced constitutions and significant differences (*P* < .05) in the degree and disability dimensions between excess and balanced constitutions. Multiple linear regression, after controlling for covariates, showed that individuals with a deficiency constitution had higher scores on duration (β = 0.505, 95% CI = 0.844–1.185), degree (β = 0.354, 95% CI = 0.029–0.68), disability (β = 0.366, 95% CI = 0.176–0.555), and distribution (β = 0.463, 95% CI = 0.185–0.742) than those with a balanced constitution. Similarly, individuals with an excess constitution had higher scores for degree (β = 0.511, 95% CI = 0.145–0.877) and disability (β = 0.294, 95% CI = 0.082–0.507) (Table 4).

**Table 3:** Scores of 5D itching dimensions in different categories of TCM constitution (n = 162).

Category of TCM constitution	Duration	Degree	Direction	Disability	Distribution
Balanced	1.10 ± 0.44	1.78 ± 0.75	3.19 ± 1.24	1.08 ± 0.14	1.24 ± 0.54
Deficiency constitution	1.61 ± 1.13*	2.1 ± 1.03	2.8 ± 1.32	1.43 ± 0.79*	1.67 ± 1.13*
Excess constitution	1.29 ± 0.86	2.26 ± 0.98*	2.94 ± 1.14	1.41 ± 0.53*	1.34 ± 0.54

TCM = traditional Chinese medicine.

* *P* < .05.

**Table 4:** The correlation of deficiency and actual constitutions with 5D itching dimensions (n = 162).

	Deficiency constitution		Excess constitution	
	β (95% CI)	*P*-value	β (95% CI)	*P*-value
Duration	0.505 (0.844 to 1.185)	.001	0.231 (0.831 to 1.203)	.164
Degree	0.354 (0.029 to 0.68)	.033	0.511 (0.145 to 0.877)	.007
Direction	−0.324 (−0.778 to 0.129)	.160	−0.245 (−0.755 to 0.265)	.344
Disability	0.366 (0.176 to 0.555)	<.001	0.294 (0.082 to 0.507)	.007
Distribution	0.463 (0.185 to 0.742)	.001	0.132 (−0.181 to 0.445)	.407

Gender, age, education, marriage, living with others, employment status and exercise regularly were adjusted in the models.

## 4. Discussion

CKD-aP is a continual problem for hemodialysis patients, causing significant physical and psychological discomfort and severely impacting their quality of life. It also leads to frequent hospital admissions and significantly increases healthcare utilization.^[24]^ The pathophysiological mechanisms underlying CKD-aP are complex and not fully understood, resulting in less effective clinical treatments.^[25]^ Thus, exploring diverse information related to disease occurrence, particularly the correlation between disease occurrence and individual differences, is crucial for providing personalized medical care. Over the past 2 decades, TCM scholars have classified TCM constitutions from a holistic and dynamic perspective to understand the correlation between specific diseases and constitution types, thereby guiding disease prevention and clinical practice more precisely.^[18,26,27]^ This study investigated the distribution of TCM body constitutions among hemodialysis patients in Macau, and explored the relationship between biased constitution types and the occurrence of CKD-aP. Due to the highly complex nature of constitutional formation, individuals may simultaneously present multiple biased constitutions. Analyzing each coexisting constitution would require a considerably larger sample size. However, with only approximately 750 hemodialysis patients in the Macau region,^[28]^ the sample size of this study was constrained. Therefore, only the primary constitution with the highest score was selected for analysis. Additionally, in terms of constitutional distribution, only 6 cases were identified as having an inherited special constitution – a number too small for independent statistical analysis. Although some studies suggest that inherited special and yin deficiency constitution are associated with skin sensitivity,^[20]^ there is no direct evidence linking inherited special constitution to the occurrence of pruritus. Consequently, the inherited special constitution was excluded from the analysis in this study.

According to the distribution of constitutions shown in Figure 1, more than half of the hemodialysis patients in Macau had a biased constitution, accounting for 53.6%. Hemodialysis is a renal replacement therapy used when chronic kidney disease progresses to end-stage renal disease typically requiring more than a decade to reach the stage where dialysis is required.^[29]^ According to TCM theory, prolonged disease progression can lead to varying degrees of Qi and blood consumption, shifting an individual’s constitution from a healthy balanced state to a biased one, thus increasing susceptibility to certain diseases, such as CKD-aP. This aligns with the findings of this study.

The statistical descriptions and univariate analysis in Table 1 indicate that imbalanced constitutions (both deficiency and excess) are associated with the occurrence of CKD-aP. Because of the limited sample size, this study, following the theory proposed by Xing et al., categorized qi deficiency, yang deficiency, and yin deficiency as deficiency constitutions, and phlegm dampness, damp heat, qi stagnation, and blood stasis as excess constitutions,^[14]^ and explored their effects on CKD-aP. After adjusting for all potential confounding factors, logistic regression analysis (Table 2) showed that the likelihood of experiencing CKD-aP was 2.2 times higher in individuals with a deficiency constitution and nearly 2.7 times higher in those with an excess constitution than in those with a balanced constitution. The TCM theory suggests that a qi deficiency constitution reflects an individual’s insufficient qi, affecting normal physiological functions. When qi deficiency is severe and cannot perform the warming function of qi, a yang deficiency constitution appears, whereas a yin deficiency constitution reflects a lack of bodily fluids such as blood and bodily fluids. The basic TCM theory states that qi can promote the movement of blood and bodily fluid, and that blood and bodily fluids can carry qi, allowing it to flow smoothly. Thus, the pathogenesis of CKD-aP in individuals with a deficient constitution may be related to insufficient qi and blood function, preventing the effective removal of toxic metabolites accumulated in the body. Previous studies have also indicated that the pathophysiological mechanisms of CKD-aP are related to the accumulation of phosphorus, serum aluminum, and various toxic metabolites in the skin.^[4]^ Regarding excess constitutions, phlegm dampness reflects poor metabolism of nutrients and poor circulation of body fluids; damp heat reflects a response of heat in addition to phlegm dampness; qi stagnation and blood stasis reflect not a lack of qi and blood but an obstruction in their circulation. These abnormal conditions further lead to the accumulation of pathological products such as lipids and glucose. Previous research on TCM constitutions also indicated that excess constitutions are related to metabolic syndrome, hyperlipidemia, coronary artery atherosclerosis, and diabetes.^[30,31]^ It is inferred that individuals with an excess constitution may experience changes in hemodynamics due to pathological factors such as hyperlipidemia and hyperglycemia, causing obstruction to blood flow entering the kidneys and reducing the excretion of metabolic toxins in the blood. In summary, although the pathogenesis of deficiency and excess constitutions differs, both may ultimately affect the removal of metabolic toxins, leading to CKD-aP.

**Figure 2.**
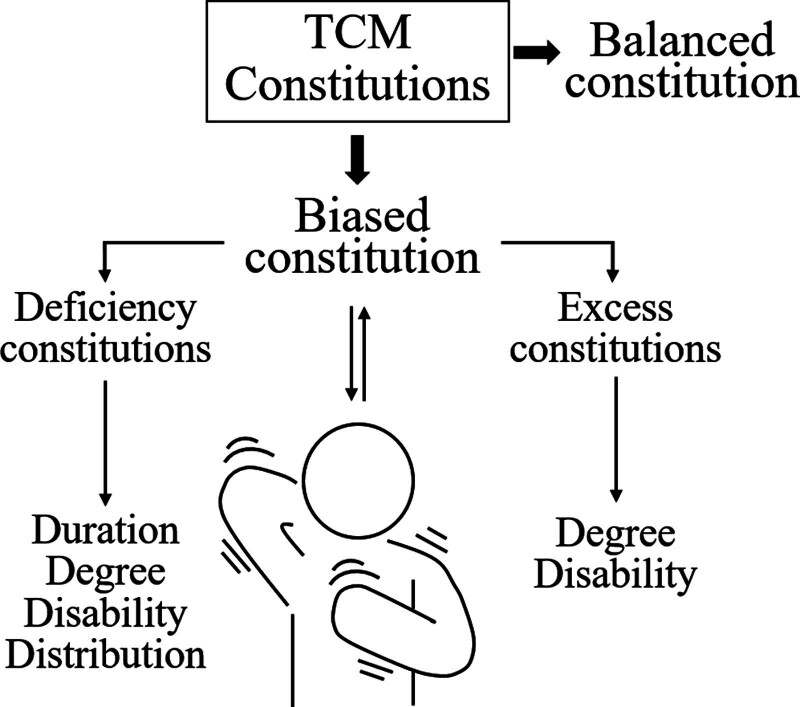
Summary of the relationship between CKD-aP and TCM constitution. The biased constitution is related to susceptibility to CKD-aP. Among them, the deficiency constitutions mainly affect the duration, degree, disability, and distribution dimensions of CKD-ap; while the excess constitutions mainly affect the degree and disability dimensions of CKD-ap.

Further analysis of the relationship between deficiency and excess constitutions and the dimensions of pruritus showed that, compared to those with a balanced constitution, individuals with a deficiency constitution primarily influenced the duration, disability, and distribution aspects of CKD-aP, whereas those with an excess constitution affected the degree and disability aspects (Fig. 2). After controlling for covariates, multivariate linear regression results indicated that individuals with a deficiency constitution had higher scores by approximately 0.35 to 0.51 times on duration, degree, disability, and distribution of pruritus than those with a balanced constitution. Those with an excess constitution had higher scores by approximately 0.29 to 0.51 times on the degree and disability dimensions compared to those with a balanced constitution. As previously mentioned, the TCM theory considers a deficient constitution to mainly reflect an individual’s insufficient qi and blood, that is, a lack of bodily energy, externally manifesting as fatigue and muscle weakness. Therefore, it is inferred that, owing to the reduced ability of qi and blood to circulate, metabolic toxins produced by the body cannot be promptly removed and continue to accumulate, causing persistent pruritus that worsens in severity and expands in the distribution area. Increasingly severe pruritus, combined with weakness caused by insufficient qi and blood; leads to disability. Excess constitutions are inferred to be due to pathological factors, such as phlegm, heat, qi stagnation, and blood stasis, which obstruct the circulation of qi and blood in the body. If these pathological factors are not eliminated, they cause increasingly severe qi and blood stagnation, worsening the severity of pruritus. If phlegm, heat, qi, and blood obstruct meridians, they may cause limited limb movement, resulting in disability. Therefore, guidelines for improving CKD-aP should consider the constitution of patients undergoing hemodialysis. For those with a deficient constitution, principles such as warming yang and benefiting qi, nourishing yin, and fluids are recommended. For those with excess constitution, principles such as removing dampness and phlegm, clearing heat and benefiting dampness, regulating qi, relieving depression, promoting blood circulation, and removing blood stasis.

This study was a cross-sectional analysis based on TCM constitutions and CKD-aP among hemodialysis patients in Macau, representing only a preliminary exploration of the relationship between patient constitutions and CKD-aP. Due to the limited sample size, it was not possible to analyze each constitutional type individually; instead, deficiencies and excess constitutions were categorized for integrated analysis. Therefore, these findings should be interpreted with caution in clinical practice. Furthermore, the mechanisms linking deficiency and excess constitutions in TCM to susceptibility to CKD-aP, as discussed, are preliminary inferences based on TCM theory. Further in-depth studies on their pathological mechanisms are warranted in the future. Future studies should design prospective experiments and reasonably increase the sample size to clarify the relationship between various TCM constitutional types and CKD-aP. Additionally, the mechanisms of CKD-aP associated with deficiency and excess constitutions, based on the TCM theory, require further research.

## 5. Conclusion

Our results indicate that both deficiency and excess constitutions among hemodialysis patients in Macau are significantly correlated with CKD-aP, suggesting that TCM body constitution factors may influence the occurrence of CKD-aP in these patients. Specifically, deficiency in constitution mainly affects the duration, degree, disability, and distribution dimensions of pruritus, whereas excess constitution mainly affects the degree and disability dimensions. Early intervention based on TCM differentiation for these constitution types, combined with appropriate treatment principles, can potentially help improve CKD-aP by adjusting imbalanced constitutions.

## Acknowledgments

We would like to thank Yingying Zhao, Wenrou Wang, Yuequn Zhou, Yuhua Tang, Huandi Liang, kain Sin, Zunhua Xie and Mio Im for the data collection.

## Author contributions

**Conceptualization:** Yao-Chen Chuang, Kun-Han Chuang.

**Data curation:** Xin Wang, Jianwei Wu.

**Formal analysis:** Jianwei Wu.

**Investigation:** Yao-Chen Chuang, Yinghong Zhang.

**Supervision:** Jianwei Wu.

**Writing – original draft:** Yao-Chen Chuang, Jianwei Wu.

**Writing – review & editing:** Yinghong Zhang, Kun-Han Chuang.
